# Effect of Ni Substitution on the Structural, Magnetic, and Electronic Structure Properties of Gd_0.4_Tb_0.6_(Co_1−x_Ni_x_)_2_ Compounds

**DOI:** 10.3390/ijms232113182

**Published:** 2022-10-29

**Authors:** Marcin Sikora, Anna Bajorek, Artur Chrobak, Józef Deniszczyk, Grzegorz Ziółkowski, Grażyna Chełkowska

**Affiliations:** 1 Institute of Physics, University of Silesia in Katowice, 41-500 Chorzów, Poland; 2 Institute of Materials Engineering, University of Silesia in Katowice, 41-500 Chorzów, Poland

**Keywords:** magnetic properties, rare earth–transition metal compounds, magnetocaloric effect, electrical resistivity, electronic structure

## Abstract

The comprehensive research of magnetic and electronic structure properties of the new class of Gd_0.4_Tb_0.6_(Co_1−x_Ni_x_)_2_ compounds, crystallizing in the cubic Laves phase (C15), is reported. The magnetic study was completed with electrical resistivity and electronic structure investigations. The analysis of Arrott plots supplemented by a study of temperature dependency of Landau coefficients revealed that all compounds undergo a magnetic phase transition of the second type. Based on magnetic isotherms, magnetic entropy change (*ΔS_M_*) was determined for many values of the magnetic field change (*μ*_0_*H*), which varied from 0.1 to 7 T. For each compound, the *ΔS_M_* had a maximum around the Curie temperature. Both values of the |*ΔS_M_^max^*| and relative cooling power *RCP* parameters increased with increasing nickel content. It is shown that structural disorder upon Co/Ni substitution influences some magnetic parameters. The magnetic moment values of Co atoms determined from different methods are quantitatively consistent. From the *M*(*T*) dependency, the exchange integrals J_RR_, J_RT_, and J_TT_ between rare-earths (R) and transition metal (T) moments were evaluated within the mean-field theory (MFT) approach. The experimental study of the electronic structure performed with the use of the X-ray photoelectron spectroscopy (XPS) was completed by calculations using the full-potential linearized augmented plane waves (FP-LAPW) method based on the density functional theory (DFT). The calculations explained experimentally observed changes in the XPS valence band spectra upon the Ni/Co substitution.

## 1. Introduction

Among magnetic materials, RCo_2_ compounds (R = rare earth) having the Laves phase structure of C15 type have attracted particular interest [1,2]. Attention to these materials is mainly due to their attractive properties, which strongly depends on the type of rare-earth element in the composition [3]. Furthermore, their simple cubic crystal structure facilitates the interpretation of the obtained results. The RCo_2_ compounds with nonmagnetic R-ions, such as Y or Lu, show enhanced Pauli paramagnetism and underwent a metamagnetic transition [1,3]. Under the influence of an external magnetic field exceeding a certain critical *H_c_* value, a transition from a paramagnetic to a ferromagnetic state occurs accompanied by the increase in initially negligible magnetic moments on the Co site, even by 0.5 *μ_B_* [4,5]. In RCo_2_ compounds with magnetic R-ions, cobalt atoms have induced magnetic moment, which occurs due to the 4*f*-3*d* interaction between localized moments of rare earth and the metamagnetic moments of cobalt [1]. Substitution of other kinds of atoms in RCo_2_ results in a new family of compounds called pseudobinaries, such as the R(Co_1−x_T_x_)_2_, the R_1−y_R′_y_Co_2_, or even four-component R_1−y_R’_y_(Co_1−x_T_x_)_2_ compounds (R′—rare earth, T—3*d* element). They are particularly interesting because of additional interactions between magnetic moments (4*f′*-4*f*, 4*f*-3*d*, 4*f′*-3*d*, and others), which may significantly alter the magnetic properties of the original material but also strongly modify their electronic structure. It is known that RCo_2_ compounds show a magnetic structure with parallel or antiparallel alignments of the magnetic moments of R and Co ions for light or heavy R elements, respectively [2]. RCo_2_ compounds are also known as materials exhibiting a significant magnetocaloric effect (MCE) [1].

Magnetic cooling based on the magnetocaloric effect is considered an alternative cooling technique to classical gas vapor cooling due to its high efficiency and environmental friendliness. The value of MCE in these compounds usually has its maximum around the Curie temperature (*T_C_*) and can be pretty high in the case of the first-order phase transition (FOPT). The magnetic materials for which *T_C_* is located near room temperature are particularly attractive due to the possibility of using them for magnetic refrigeration in consumer devices. Recently, we reported on the comprehensive experimental and theoretical studies of magnetic and electronic structural properties of the Gd_0.4_Tb_0.6_Co_2_ compound [6], for which the *T_C_* was earlier determined to be near room temperature [7]. We have shown that this compound is a material with minimal hysteresis losses and reasonable relative cooling power (RCP) parameter values at room temperature, which qualifies it for use in magnetic refrigerators. Considering the Gd_0.4_Tb_0.6_Co_2_ as a basis for the new class of four-component compounds, we have studied the Gd_0.4_Tb_0.6_(Co_1−x_Ni_x_)_2_ system. We investigated its electronic structure, particularly in the valence band range, essential for magnetic properties. The study of the electronic structure was conducted experimentally and theoretically. Our results are entirely new for the system under investigation. Moreover, using the two-sublattice model, in the mean-field theory (MFT) approximation, the exchange integrals J_RR_, J_RCo_, and J_CoCo_ were evaluated. Within this theory, we determined the magnetic moment of Co atom, *μ*_Co-MFT_, and compared it with its values obtained by other methods.

## 2. Results and Discussion

### 2.1. Crystal Structure

**Figure 1:**
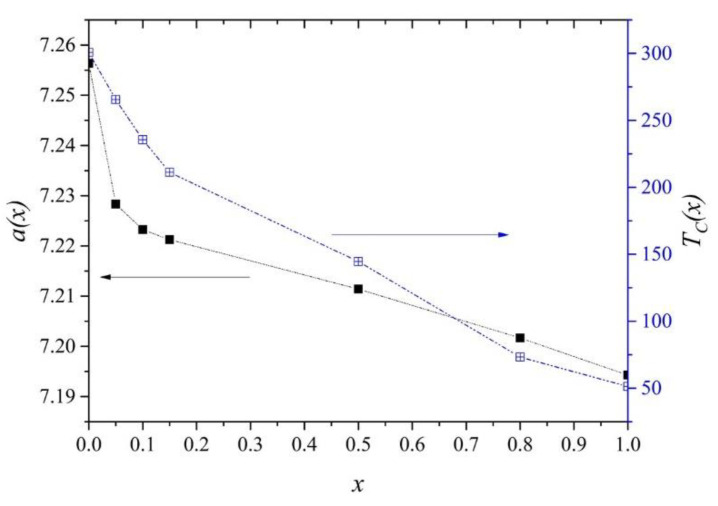
Lattice parameter *a* as a function of *x* (left axis). Curie temperature *T_C_* derived from magnetic measurements, discussed in Section 2.2 (right axis).

The crystal structure of all analyzed compounds was refined using the Rietveld method, and the analysis was carried out using noncommercial Maud software ver. 1.6.4 (Pandata, Berlin, Germany) [8,9]. The study showed that the samples with x = 0.00, 0.05, 0.10, 0.15, and 0.50 crystallized in the MgCu_2_ type of structure (Fd-3m space group) and were free from undesirable magnetic impurities. In contrast, the sample with x = 0.80 contained additionally about 3.5% of the Gd_40_Tb_60_ phase. The sample with *x* = 1.00 crystallized in the superstructure of MgCu_2_ with double cell parameter *a* and contained two additional undesirable magnetic phases (Tb_2_O_3_, 9.8%) and TbNi (7.2%). With increasing Ni content, the value of the *a*, which for the Gd_0.4_Tb_0.6_Co_2_ compound was equal to 7.256 Å ± 0.001 Å, first decreases rapidly for *x* less than 0.15 and then for higher concentrations decreases following Vegard’s law (Figure 1, left axis). The cell parameter generally lowers because nickel atoms have smaller ionic radii than cobalt atoms. The deviation from Vegard’s law, visible for small Ni concentration (<0.15) can be attributed to magneto-volume effects, as the measurements (at 294 K) have been carried out not far away from *T_C_*. A similar situation was observed in Gd(Co_1−x_Ni_x_)_2_ compounds [10,11].

### 2.2. Magnetic Properties

**Figure 2:**
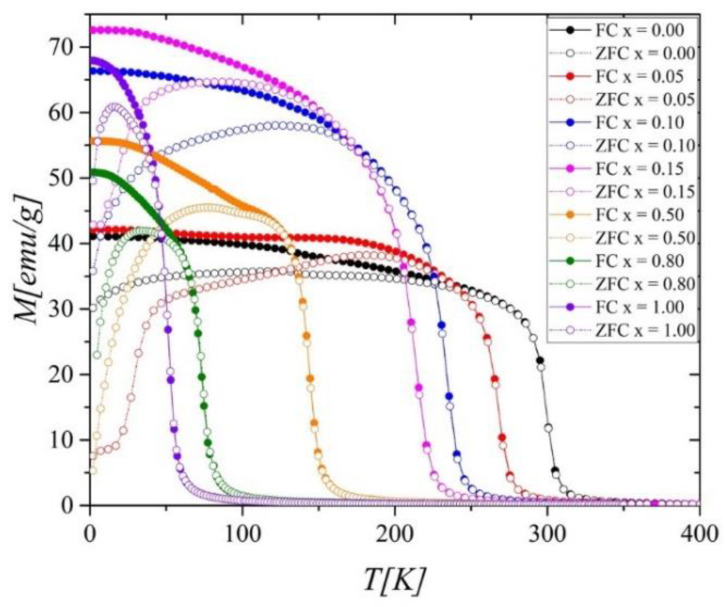
Magnetization *M* versus temperature, measured in FC and ZFC mode (*M*_FC_ and *M*_ZFC_) at a magnetic field of 0.1 T.

The temperature dependence of the magnetization *M*(*T*) measured in zero-field cooling (ZFC) and field cooling (FC) mode at the external magnetic field of 0.1 T is shown in Figure 2.

**Figure 3:**
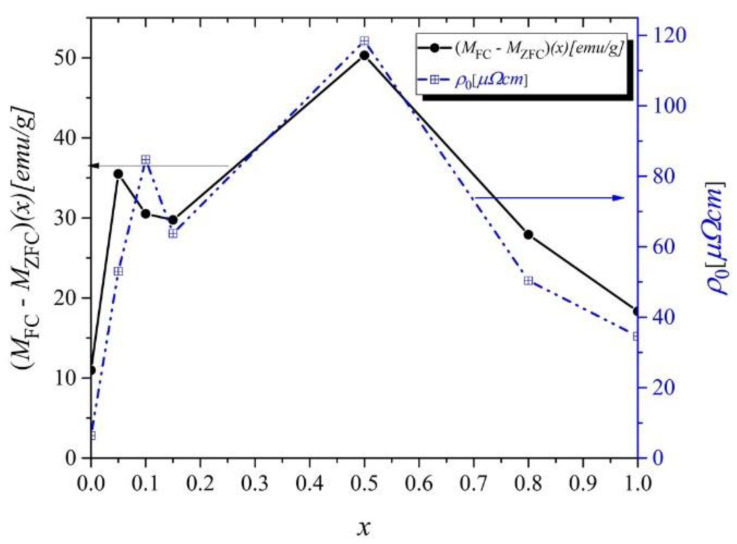
Difference between the *M*_FC_ and *M*_ZFC_ values at 2 K (left axis). Residual resistivity (*ρ*_0_) vs. concentration *x*, discussed in Section 2.4 (right axis).

The thermomagnetic curves exhibit irreversible behavior in the low-temperature range. The difference between the *M*_FC_ and *M*_ZFC_ at 2 K changes with Ni concentration and is most significant for *x* = 0.5 (Figure 3).

Several reasons may be responsible for the observed *M*_FC_*-M*_ZFC_(*x*) differences. Some of these include structural and magnetic disorders; others point to spin–orbit coupling changes that influence magnetic anisotropy, the domain wall pinning effect, or the impact of the crystal field [12,13,14,15,16,17]. According to our results discussed later, we are rather convinced that the indicated difference is due to structural and magnetic disorders.

**Figure 4:**
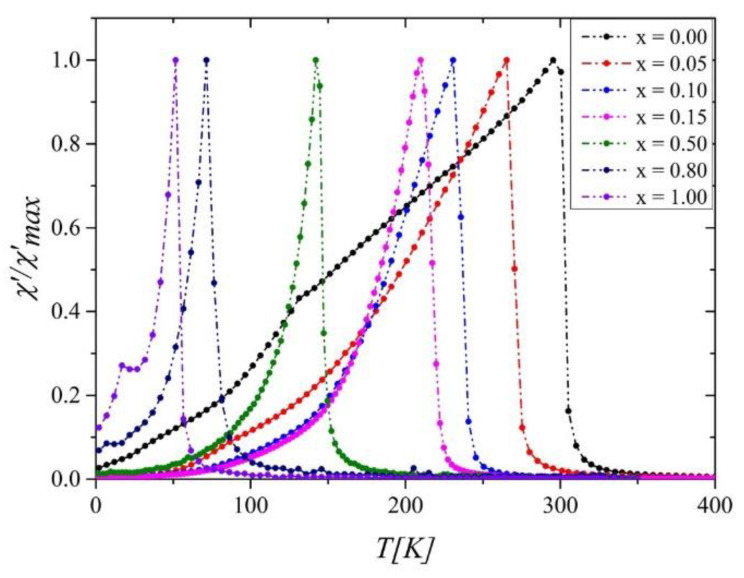
The real part of the *AC* magnetic susceptibility *χ^’^* normalized against the maximum value of *χ*^’^_max_ for the Gd_0.4_Tb_0.6_(Co_1−x_Ni_x_)_2_.

The real parts of the magnetic susceptibility *AC* (*χ^’^*(*T*) in Figure 4) showed characteristic peaks at magnetic transition temperatures *T_C_*. The values of *T_C_* determined from *χ^’^* 1 and from minima of *dM*/*dT* in both ZFC and FC modes are in perfect agreement, showing a significant decrease with increasing Ni content (Figure 1, right axis). This effect can be related to the dilution of the Co subsystem when replacing Co atoms with weaker magnetically Ni atoms. Similar behavior was observed in many Laves phase compounds doped by nickel [18,19]. The noticeable consistency of *a*(*x*) and *T_C_*(*x*) plots confirm that *T_C_* and magnetic properties have a strong lattice volume dependence in these compounds [10,20].

**Figure 5:**
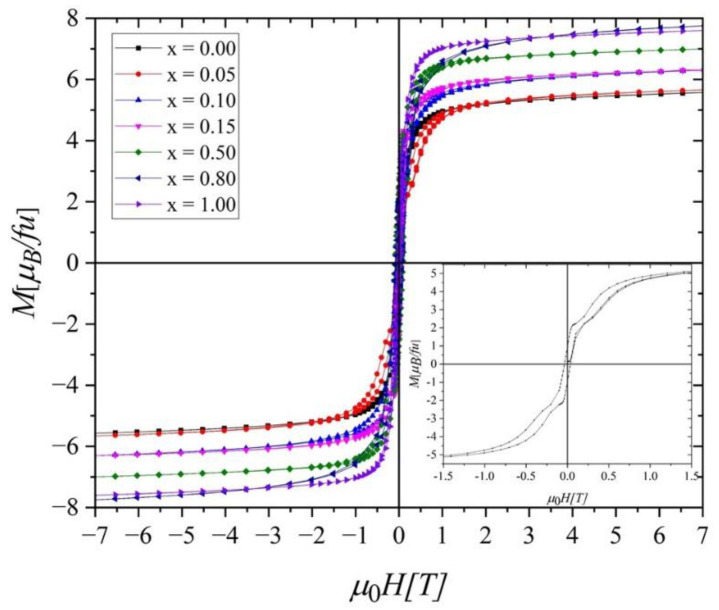
Hysteresis loops measured at 2 K. The inset shows *M*(*μ*_0_*H*) in the low magnetic field values range for the sample with *x* = 0.05.

The hysteresis loops measured at 2 K, 25 K, 50 K, 100 K, and 300 K show minimal hysteresis losses for all investigated samples (Figure 5). Moreover, no saturation has been observed even at *μ*_0_*H* = 7 T. Using *M*(*H*) data at 2 K, the saturation magnetization (*M_S_*) was determined from extrapolation to zero of 1/*H* in the *M* vs. 1/*H* dependence. The value of *M_S_* grew monotonically with the nickel content, except for the sample with *x* = 1.0 (Table 1). Using the obtained values for *M_S_*, we estimated the magnetic moment of the 3*d* sublattice (2 *μ*_3*d*_) versus *x,* applying the formula *M_S_* = 0.4 *μ_Gd_* + 0.6 *μ_Tb_* + 2 *μ*_3*d*_ with *μ*_3*d*_ = (1 − *x*) *μ_Co_* + *x μ_Ni_*, *μ_Gd_* = 7 *μ_B_,* and *μ_Tb_* = 9 *μ_B_*. For *x* = 0 (*M_S_* = 5.86 *μ_B_*), the procedure gave *μ_Co_* = −1.17 *μ_B_*, which is in accordance with earlier results in these kinds of compounds [6,11]. The negative sign indicates the antiparallel alignment of the R and Co moments. With the growing content of Ni, which is nearly nonmagnetic [11,21], the 3*d* sublattice moment 2 *μ*_3*d*_ decreases (Table 1). Because *μ*_3*d*_ and *μ_R_* are oriented antiparallel, it yields the observed increase in *M_S_*(*x*).

All the hysteresis loops show a symmetric course after changing the external magnetic field direction; however, for the sample with *x* = 0.05, a slight deformation was observed in the *M*(*H*) chart, suggesting a coexistence of hard and soft magnetic phases (inset of Figure 5). Materials showing such behavior are known as exchange spring magnets [22,23]. In the case of our sample, it may be related to the presence of a foreign magnetic phase in tiny amounts below the XRD detectability.

**Figure 6:**
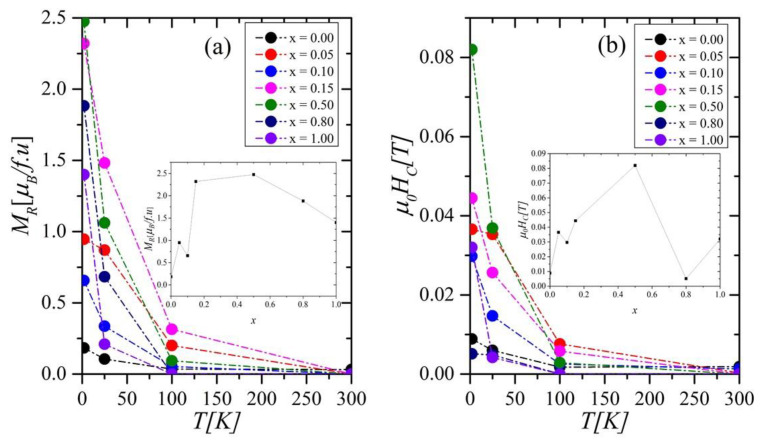
The residual magnetization *M_R_* (**a**) and coercive fields *H_C_* (**b**) versus *T* and chemical composition *x* in the Gd_04_Tb_0.6_(Co_1x_Ni_x_)_2_ compounds. The insets in Figure (**a**) and (**b**) present the concentration dependence of *M_R_* and *H_c_*, respectively.

Figure 6a,b shows the residual magnetization *M_R_* and coercive fields *H_c_* as a function of *T* in the Gd_0.4_Tb_0.6_(Co_1−x_Ni_x_)_2_ system. As can be seen, with the increase in the nickel content, the *H_c_* measured at 2 K grows up to the maximum value of 0.083 T for *x* = 0.5, then decreases to 0.005 T for *x* = 0.8 (inset in Figure 6b). A similar trend, with a maximum at the same Ni concentration, was observed for *M_R_*(*x*) (inset in Figure 6a). A slight rise of the *H_c_* value for *x* = 1.0 might be related to additional magnetic phases in the sample. The *H_c_*(*x*) dependence perfectly reflects the *ρ*_0_(*x*) dependence (which will be discussed later) and is consistent with the *M*_FC_*-M*_ZFC_ one (Figure 2). Thus, all these dependencies appear to have a common origin: the structural disorder and magnetocrystalline anisotropy influence that occur with doping.

**Figure 7:**
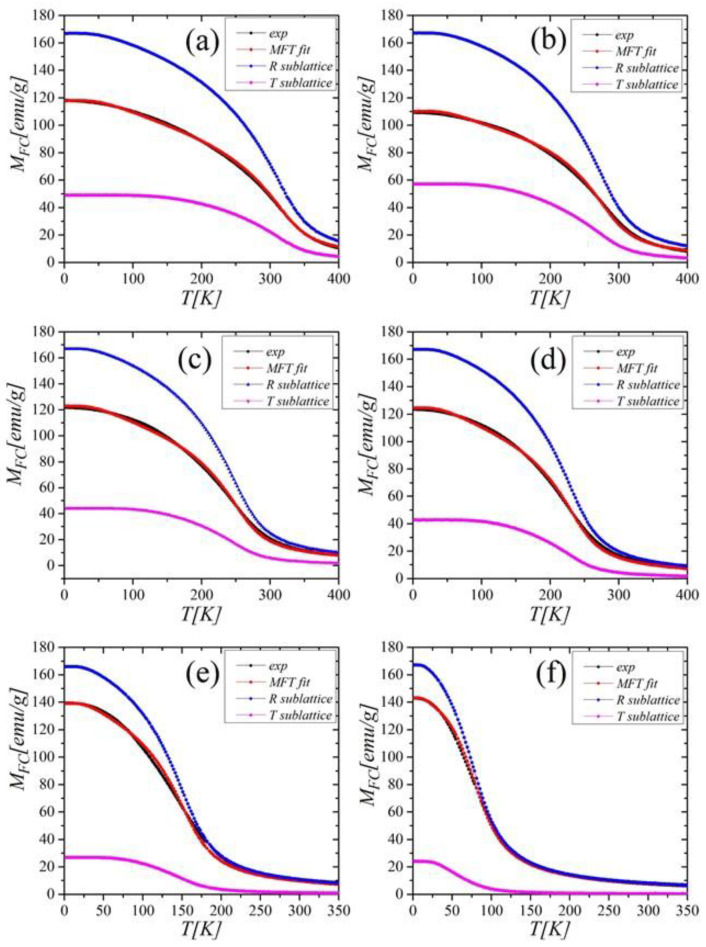
The *M*_FC_(*T*) dependence at *μ*_0_*H =*5 T (*exp*) and the results of MFT analysis for Gd_0.4_Tb_0.6_(Co_1−x_Ni_x_)_2_ compounds with concentrations x = 0.00; 0.05; 0.10; 0.15; 0.50; and 0.80 (figures (**a**–**f**), respectively).

The magnetic properties of materials containing R (4*f*) and T (3*d*) elements are determined by exchange interactions between spins of constituent atoms. The strength of these interactions is described by the exchange integrals J_RR_, J_TT_, and J_RT_ (= J_TR_). To estimate the 3*d*-3*d* and 3*d*-4*f* exchange interactions in Gd_0.4_Tb_0.6_(Co_1−x_Ni_x_)_2_ compounds, we considered its magnetic structure as consisting of two magnetic sublattices formed by R(Gd/Tb) and T(Co/Ni) moments. We performed the estimation by applying the approach described in detail in [6,24,25]. In the evaluation, we used the *M*(*T*) dependence under the magnetic field of 5 T, which was high enough to avoid the influence of domain effects and achieve a relatively high saturation (black curves in Figure 7). Table 2 shows the results of exchange coupling integrals calculated under the assumption that the average coordination numbers of RR (Z_RR_), RT (Z_RT_), TR (Z_TR_), and TT (T_TT_) are equal: 4, 12, 6, and 6, respectively [26].

The results showed that the R–R interactions remain constant for the samples with 0≤ *x* ≤ 0.5 (Table 2). The lower values of exchange interactions obtained for *x* = 0.8 and *x* = 1.0 may be related to the presence of foreign magnetic phases in these samples and the superstructure of the last one. As Ni concentration increases, R–T and T–T interactions weaken, but the latter is almost one order of magnitude higher than the others. The negative sign of J_RT_ means the antiparallel coupling between R and T moments. Taking *μ_Gd_* = 7 *μ_B_* and *μ_Tb_* = 9 *μ_B_,* we determined the average magnetic moment per 3*d* sublattice (2 *μ*_3*d-MFT*_), as a function of *x* (Table 2). The obtained values are slightly higher than those derived from *M_S_* (Table 1), but the same decreasing trend is preserved. One has to note that *M_s_* was determined from the magnetization curve up to 7 T, while for MFT analysis, we used magnetization curves measured at 5 T. Based on the obtained results, we claim that the weakening of R–T and T–T interactions is responsible for decreasing *T_C_* and the magnetic moment of the 3*d* sublattice in the investigated system.

The Arrott plots (not shown here) indicate that the investigated compounds undergo a phase transition of the second-order (SOPT). To confirm the observation, we determined the type of phase transition using the Landau expression for the magnetic free energy (*F*) [14]:

(1) $$F=\frac{1}{2}a\left(T\right)M^{2}+\frac{1}{4}b\left(T\right)M^{4}+\frac{1}{6}c\left(T\right)M^{6}-\mu_{0}HM$$

The temperature dependence of Landau coefficients *a*(*T*), *b*(*T*)*,* and *c*(*T*) are usually used to identify the type of phase transition. They are accessible through the following relation between *M* and *H* [15]:

(2) $$\;\mu_{0}H=a\left(T\right)M+b\left(T\right)M^{3}+c\left(T\right)M^{5}$$

Essentially, the order of the magnetic transition is governed by the sign of *b*(*T*). The FOPT takes place if *b*(*T_C_*) *<* 0, while the SOPT occurs when *b*(*T_C_*) ≥ 0 in the vicinity of *T_C_* [15]. The coefficients were determined by fitting Equation (2) to magnetic isotherms *μ*_0_*H*(*M*) (not shown here).

**Figure 8:**
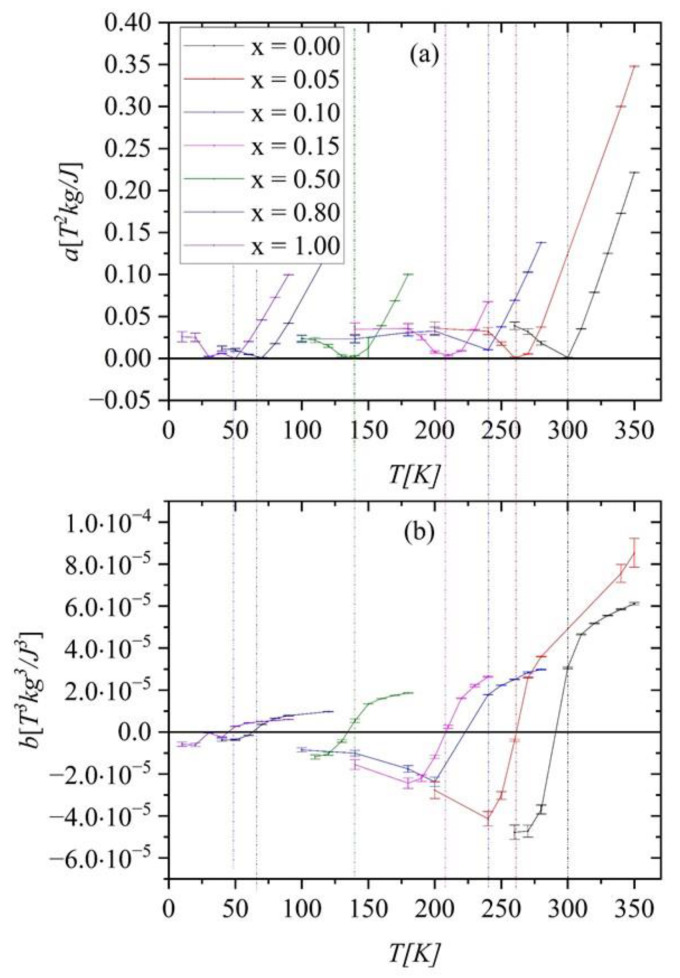
The temperature dependence of Landau coefficients *a*(*T*) and *b*(*T*)—figures (**a**) and (**b**), respectively. Vertical lines indicate transition temperatures (*T_C_*) for alloys with different *x*.

As shown in Figure 8, the *a*(*T*) exhibits a minimum nearby *T_C_*. The *b*(*T*) parameter is positive, proving that we are dealing with SOPT and confirming the result obtained from Arrott’s plots.

### 2.3. Magnetocaloric Properties

In materials showing SOPT, the magnetic entropy change is lower than in those with FOPT. However, the former usually has a broader working temperature range. This property of SOPT is significant for potential applications, e.g., in magnetic refrigerators [16].

To calculate the magnetic entropy change *ΔS_M_* based on the measured magnetic isotherms, we used Maxwell’s relation:

(3) $$\Delta S_{M}\left(M,H\right)=\int_{H_{0}}^{H_{1}}{\left(\frac{\partial M}{\partial T}\right)}_{H}dH,$$

where *H*_0_ and *H*_1_ are the initial and final magnetic fields in the above formula, respectively.

**Figure 9:**
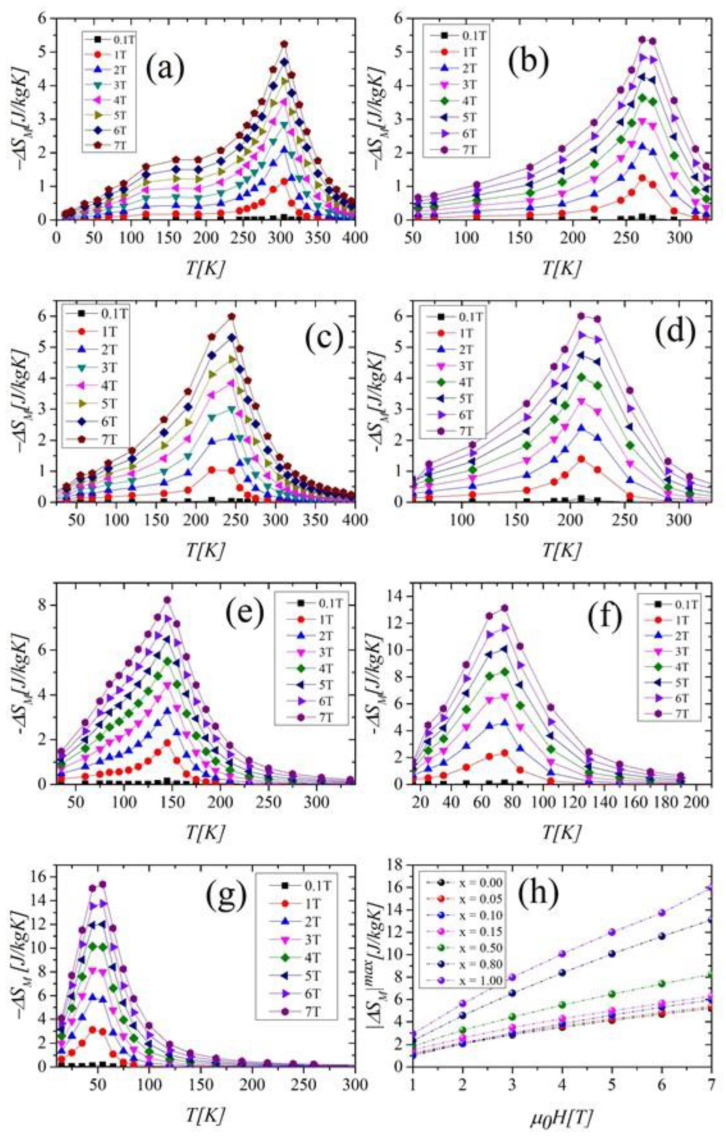
Magnetic entropy changes −*ΔS_M_* as a function of temperature for samples with *x* = 0.00 (**a**), 0.05 (**b**), 0.10 (**c**), 0.15 (**d**), 0.50 (**e**), 0.80 (**f**), 1.00 (**g**). Variation of the maximum of the entropy changes |*ΔS_M_*|^max^ with the growth of a magnetic field (**h**).

As can be seen in Figure 9, the maxima of the entropy changes −*ΔS_M_* occur near *T_C_*, which is typical for compounds exhibiting SOPT. It is noteworthy that the value of |*ΔS_M_*|^max^ increases with growing nickel content (Figure 9h, Table 3). In addition, we found that the *ΔS_M_* curves are only symmetrical to *T_C_* in a limited temperature range (*T_C_ ±* 50 K). For the compound with *x* = 0.0, this effect may be related to a partial reorientation of the Tb spin toward the easy axis direction [6,27]. For *x* ≠ 0, this may also be influenced by the magnetic disorder. The height and the width of curves increase with the growth of the magnetic field for all compounds. As a result, the value of *δT_FWHM_* (called the operating temperature and defined as the full width at half-maximum of the −*ΔS_M_* peak) also increases with *μ*_0_*H.*

**Figure 10:**
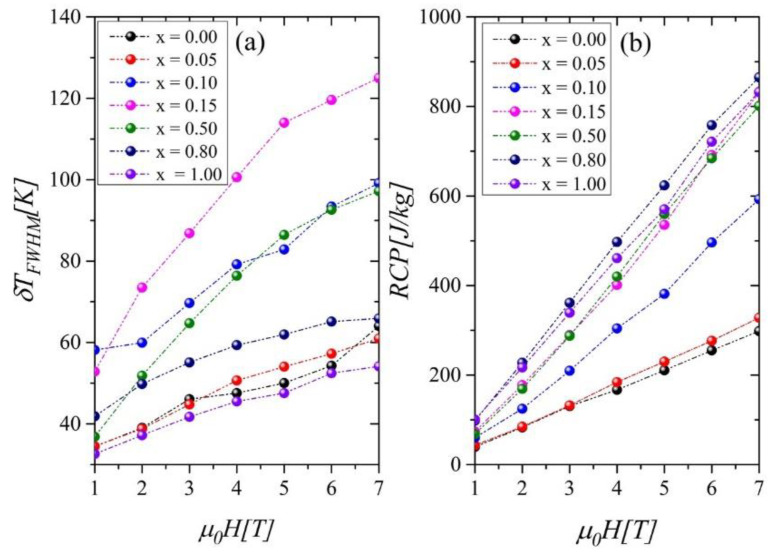
Operating temperatures *δT_FWHM_* (**a**) and *RCP* parameters as a function of a magnetic field (**b**).

The most significant rise of the *δT_FWHM_* values occurs for the compound with *x* = 0.15 (Figure 10a). To assess the cooling efficiency, we calculated the relative cooling power parameter *RCP* using the formula:

(4) $$RCP={\left|\Delta S_{M}\right|}^{max}\delta T_{FWHM}$$

The cooling efficiency was also evaluated by using the value of refrigerant capacity (*RC*), defined as an amount of the heat that can be transferred from the cold end (at *T_cold_*) to the hot end (at *T_hot_*):

(5) $$RC=\int_{T_{cold}}^{T_{hot}}{\left|\Delta S_{M}\right|}^{max}dT.$$

The *RCP* parameters vs. *μ*_0_*H* show the most rapid increase for Ni-rich samples, in which the presence of foreign phases was detected (Figure 10b). The |*ΔS_M_*|*^max^, RC, RCP,* and *δT_FWHM_* parameters for all investigated samples are collected in Table 3.

### 2.4. Electrical Resistivity

**Figure 11:**
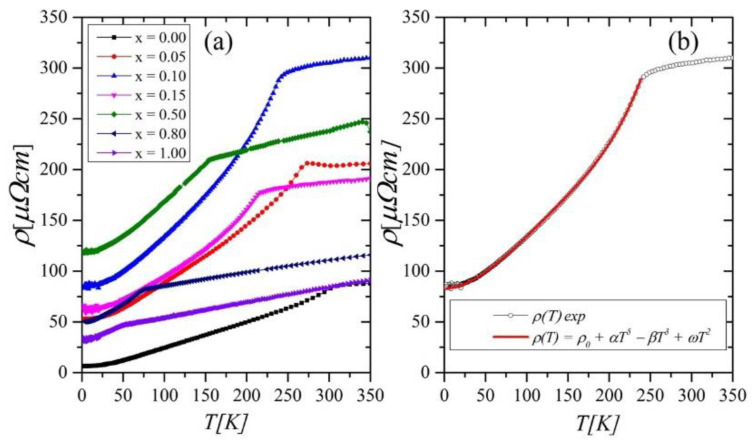
(**a**) Electrical resistivity *ρ*(*T*) for the Gd_0.4_Tb_0.6_(Co_1−x_Ni_x_)_2_ compounds, (**b**) the fit of the experimental *ρ*(*T*) for *x =* 0.10, according to Formula (7).

The electrical resistivity as a function of temperature for the investigated compounds is presented in Figure 11. We analyzed experimental data using Matthiessen’s rule, which combines individual contributions to the resistivity according to [28]:

(6) $$\rho \left(T\right)=\rho_{0}+\rho_{ph}\left(T\right)+\rho_{s-d}\left(T\right)+\rho_{e-e}\left(T\right).$$

In the formula, *ρ*_0_ indicates residual resistivity, which is temperature independent but strongly depends on the crystal structure [28]. *ρ_ph_*(*T*), *ρ_s-d_*(*T*) and *ρ_e-e_*(*T*) are the contributions from electron–phonon scattering, *s–d* scattering and electron–electron scattering, respectively [29,30].

Considering the temperature dependence of the individual contributions, Equation (6) for low temperatures takes the form:

(7) $$\rho \left(T\right)=\rho_{0}+\alpha T^{5}-\beta T^{3}+\omega T^{2}$$

All the studied compounds show an evident change in the slope of the *ρ*(*T*) curves at the Curie temperatures *T_CR_*, which were determined from the *dρ*/*dT* (not presented here). The values of *T_CR_* are in reasonable agreement with those obtained from magnetic studies. The experimental data of *ρ*(*T*) fitted very well with Formula (7) below *T_CR_,* as shown in Figure 11b for the sample with x = 0.10. The resulting parameters *α, β, ω,* residual resistivity *ρ*_0*,*_ and *T_CR_* are collected in Table 4. One can notice that the amplitudes of individual contributions to the resistivity (*α, β, ω*) show an increasing tendency with the growth of nickel content. The rise of the value of the *β* parameter, which reflects *s–d* scattering, is due to the substitution of Ni for Co and increasing the number of 3*d* electrons in the band, which may influence the scattering centers of the *d* states. An increasing number of 3*d* electrons can also lead to enhanced electron–electron scattering, which was reflected in the growth of the *ω* parameter. The behavior of residual resistivity as a function of nickel concentration *ρ*_0_(*x*) satisfies the Nordheim rule [31]. Namely, the highest value of the *ρ*_0_ is reached for *x* = 0.50 (Figure 3 right axis). Similar behavior is often observed in alloys during doping. The substitution of one type of atom by others generally leads to a disorder of the compound’s crystal structure, which manifests as an increase in residual resistivity. It is worth noting that magnetic parameters, such as *H_c_*(*x*) and (*M*_FC_*-M*_ZFC_) (*x*)*,* also showed a similar behavior as *ρ*_0_(*x*)*,* which indicates that structural disorder may be responsible for the observed trends.

### 2.5. X-ray Photoelectron Spectroscopy (XPS)

**Figure 12:**
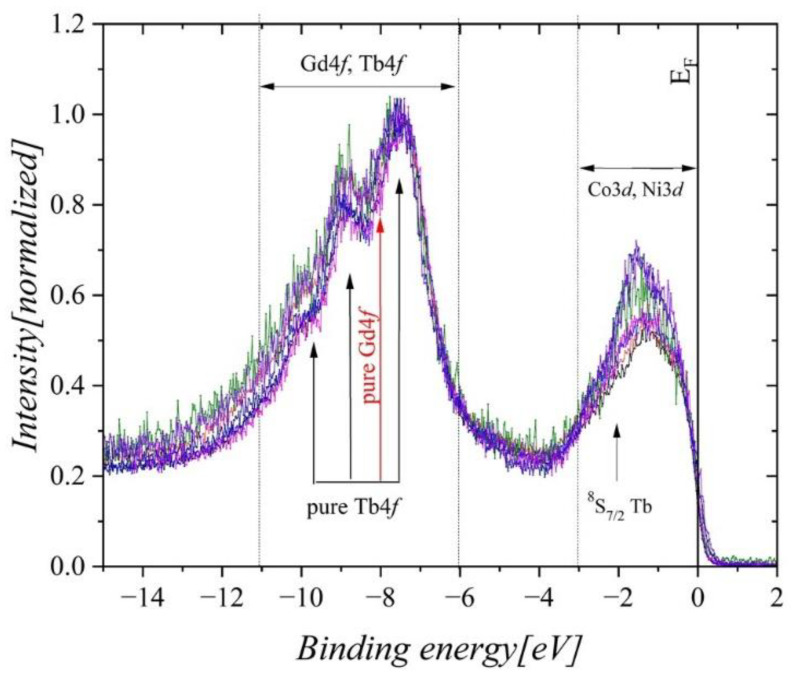
The XPS valence band of Gd_0.4_Tb_0.6_(Co_1x_Ni_x_)_2_. The spectra were normalized to the maximum intensity in this energy range. Vertical arrows show the positions of all spectral lines observed in elemental Gd, Tb, Co, and Ni.

Figure 12 presents the XPS valence band spectra of the Gd_0.4_Tb_0.6_(Co_1−x_Ni_x_)_2_ compounds in the binding energy range from −15 eV to 2 eV. In the case of pure rare earth elements, the Gd4*f* states are visible at −8 eV, while the Tb4*f* ones form the multiplet consisting of several lines located at −10.2 eV, −9.1 eV,−7.4 eV, −2 eV [32]. In the compounds under investigation, the first three Tb4*f* contributions and the Gd4*f* one overlap to form a wide band, not dependent on the Ni concentration. Bands near the Fermi level (*E_F_*) are dominated by Co3*d* and *Ni*3*d* states that form one shared 3*d* band, and they overshade the ^8^*S*_7/2_ contributions of Tb occurring at −2 eV.

**Figure 13:**
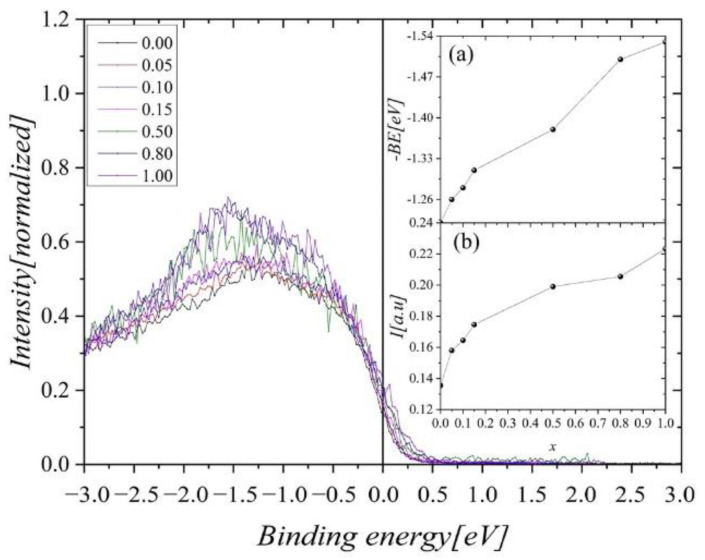
The XPS valence band of Gd_0.4_Tb_0.6_(Co_1x_Ni_x_)_2_ near the Fermi level. Inset (**a**) shows the position of the maximum of 3*d* states contribution and inset (**b**) presents the intensity at the Fermi level, both as a function of Ni concentration.

An evident change in the shape of the 3*d* valence band is observed when nickel content increases (Figure 13). In the energy range from 0 eV to −3 eV, the band intensity increases with growing Ni content. Slightly less increment is visible at the Fermi level (inset (b) in Figure 13). Simultaneously, a shift in the maximum intensity of the 3*d* states toward higher binding energies is observed (inset (a) in Figure 13).

**Figure 14:**
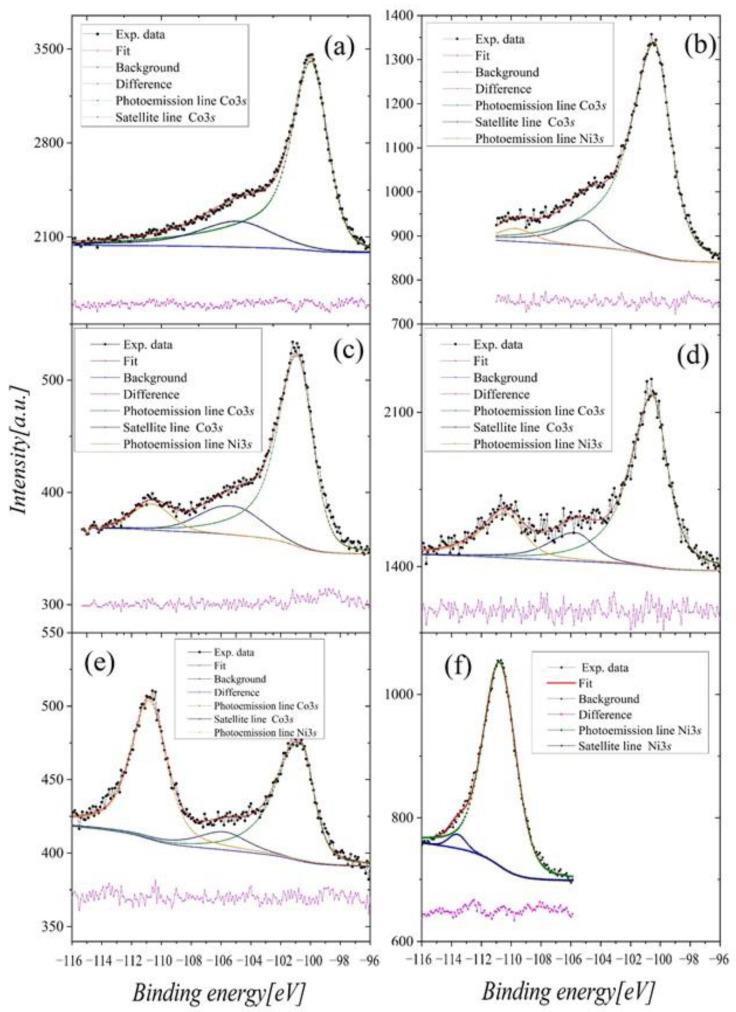
The splitting of the Co3*s* and Ni3*s* lines in Gd_0.4_Tb_0.6_(Co_1x_Ni_x_)_2_. The plots (**a**)–(**f**) concern samples with concentrations *x* = 0.0, 0.05, 0.10, 0.15, 0.50 and 1.0, respectively.

From the multiplet splitting of the Co3*s* (Figure 14), we obtained information about the magnetic moments *μ_Co_*. The multiplet splitting of the 3*s* spectra of 3*d* metals occurs due to the exchange interaction between unfilled 3*d* and 3*s* (ionized) shells [33]. As a result, two final states are observed, and the equation gives the intensity relation of these two peaks [34]:

(8) $$\frac{I_{1}}{I_{2}}=\frac{S+1}{S}$$

Here, *S* is the spin of the unpaired 3*d* electrons, *I*_1_ and *I*_2_ are intensities of the main and the satellite 3*s* line, respectively. The magnetic moment of the Co can be estimated using Formula [35]:

(9) $$\mu_{Co}=2\mu_{B}\sqrt{S\left(S+1\right)}$$

The *μ_Ni_* was similarly estimated only for *x* = 1, since the Ni 3*s* satellite lines are hardly resolved in XPS spectra for lower Ni concentration.

The *I*_1_/*I*_2_ ratio (Equation (8)) was evaluated by fitting the Co3*s* and Ni3*s* lines applying iterative Shirley background and using the combination of Gaussian–Lorentz curves. This procedure enabled us to reproduce the main shape line and obtain the intensity of the individual contributions [36] (Figure 14). Using the above-described approach, we got the following values of the *μ_Co_*: 0.95 *μ_B_,* 0.80 *μ_B_,* 1.02 *μ_B_,* 0.97 *μ_B_* and 0.98 *μ_B_* for *x =* 0.0, 0.05, 0.10, 0.15 and 0.50, respectively. The values of *μ_Co_* deviate slightly from those obtained from the saturation magnetization and the MFT calculations. However, we must remember that the XPS experiment was performed in different conditions than the previous ones (room temperature, no magnetic field). Furthermore, since the intensity of the Co3s line became weaker with increasing nickel content, the resulting moments were subjected to greater error. The magnetic moment of Ni estimated from the fit presented in Figure 14f was equal to 0.05 *μ_B_.* It is worth noting that the result confirms the rightness of our earlier assumption about the small magnetic moment on nickel.

### 2.6. Ab Initio Results

Quantitative magnetic results of ab initio calculations ($M_{S}$ and *μ*_3*d*_) are shown in Table 1. The magnetization $M_{S}$ for compositions Gd_0.375_Tb_0.625_(Co_1−x_Ni_x_)_2_ was estimated using the relation: $M_{S}=0.375\cdot {\bar{\mu }}_{Gd}+0.625\cdot {\bar{\mu }}_{Tb}+2\cdot \left(\left(1-x\right)\cdot {\bar{\mu }}_{Co}+x\cdot {\bar{\mu }}_{Ni}\right),$ where the *z*-projected average R4*f* magnetic moment was evaluated using the formula $\mu_{R}^{4f}=g_{J}\cdot \left(L+S\right)$ [35]. The values of $\mu_{Co}$ and $\mu_{Ni}$ were taken from ab initio calculations. Since within the superstructures, Gd_3_Tb_5_Co_16-n_Ni_n_ (n = 0, 2, 4, 6, 8, 16) applied in calculations, each component atom occupies several Wyckoff positions with specified multiplicity, and the ${\bar{\mu }}_{Co}$, ${\bar{\mu }}_{Ni}$ were obtained by averaging local (Co and Ni) atomic moments over Wyckoff positions. Due to the lack of ab initio results for the orbital angular momentum of R, we assumed L = 3, S = 3, and L = 0, S = 7/2, corresponding to Tb and Gd in an ionic state with valency 3^+^ [35].

The calculated T and R local magnetic moments align oppositely in agreement with experimental observations. Furthermore, the dependence of calculated magnetization *M_S_* on Ni content quantitatively follows the determined experiment. Detailed analysis displayed that with increasing Ni content in the range x = 0.0–0.5, the magnitudes of both ${\bar{\mu }}_{Co}$ and ${\bar{\mu }}_{Ni}$ decrease in the range of 1.28–1.21$\mu_{B}$ and 0.25–0.21$\mu_{B}$, respectively. Only in the Ni-rich Gd_0.375_Tb_0.625_Ni_2_ compound the average ${\bar{\mu }}_{Ni}$ has dropped to 0.13 $\mu_{B}$. For the considered Ni concentrations, the magnetization $M_{S}$ increases following the simple magnetic dilution in the Co-Ni sublattice, where the higher magnetic moment of Co is replaced by the lower one of Ni. The linear fit to calculated *M_S_* yields the relation *M_S_*(*x*) *=* 2.104 *x +* 5.942(R^2^ = 0.998). The values of *M_S_* estimated with the formula are compared with the experimental ones in Table 1. Some discrepancy between experimental data and theoretical estimates occurs for x = 0.1 and x = 0.15, where calculations underestimate $M_{S}$ but at most by a few percent. It can be related to the atomic disorder, as evidenced by the residual resistivity measurements (see Figure 3). The more significant deviations occur between the calculated average $\mu_{3d}$ and derived from the MFT approach (Table 1). Although both results show diminishing 3*d* lattice magnetization, the ab initio calculations give the $\mu_{3d}$ moment systematically overestimated.

**Figure 15:**
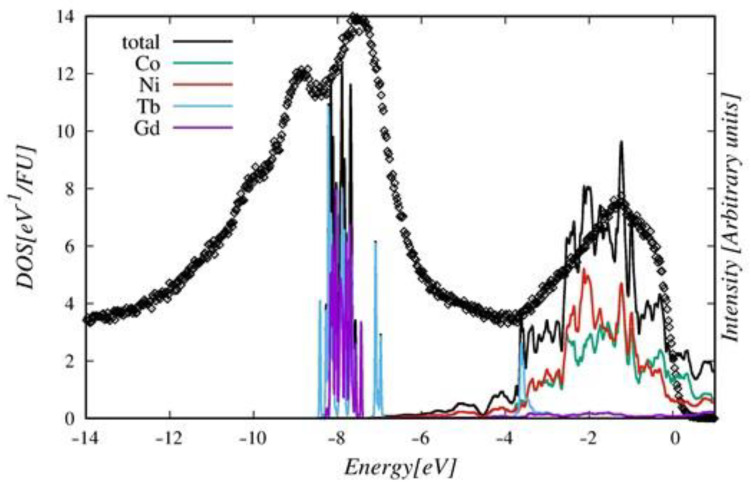
Atomic resolved and total DOS in Gd_0.4_Tb_0.6_(Co_0.5_Ni_0.5_)_2_ (solid lines), compared with the scaled XPS spectra (black points).

To get insight into the microscopic origin of the changes of XPS spectra with increasing Ni content in the Gd_0.4_Tb_0.6_(Co_1−x_Ni_x_)_2_ series, we performed a detailed analysis of the electronic densities of states (DOS) obtained from ab initio calculations. Figure 15 confirms an almost strict correspondence between the shape of the XPS spectrum and the calculated DOS in Gd_0.4_Tb_0.6_(Co_0.5_Ni_0.5_)_2_. Noticeable inconsistencies between the DOS and the XPS spectrum exist near the Fermi level and in the range of the R4*f* band. We presented the explanation of the discrepancies in [6].

**Figure 16:**
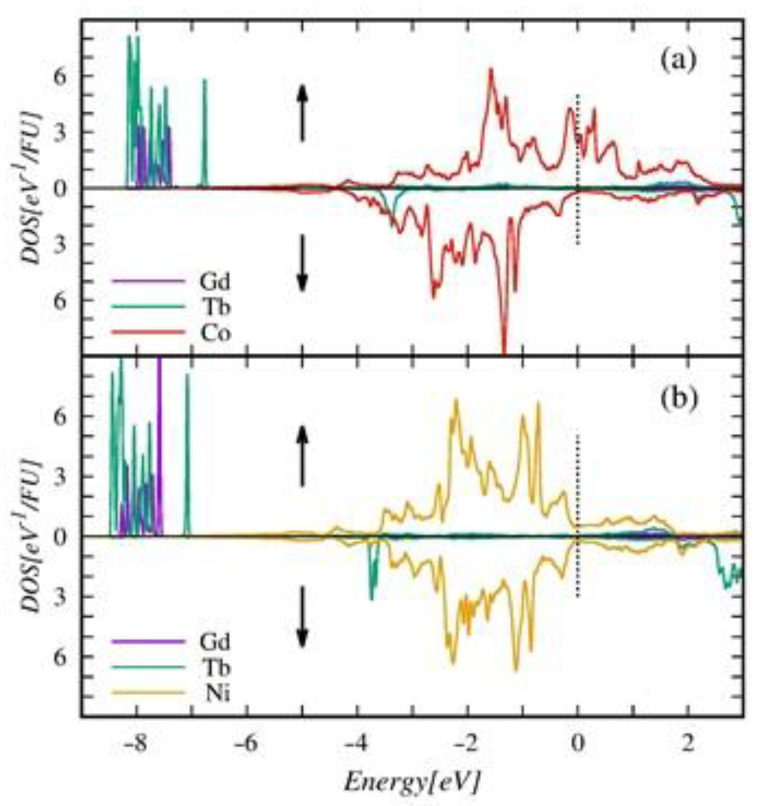
Spin resolved atomic contributions to the total density of states (DOS) calculated for end compounds: Gd_0.375_Tb_0.625_Co_2_—(**a**) and Gd_0.375_Tb_0.625_Ni_2_—(**b**). The energy scale zero is shifted to the Fermi level ε_F_ (vertical dot lines). The arrows distinguish the spin orientations of electronic states.

In the observed XPS spectra related to the 3*d* band (Figure 13a), the intensity increases and the maximum shifts simultaneously to higher BE energy with rising Ni content. To understand the reasons for such behavior in the first stage, we analyze the DOS of endpoint compounds presented in Figure 16.

The valence band structure of the endpoint compounds comprises the 4*f* states of R atoms, lying deeply (around −8 eV) below Fermi energy ($\varepsilon_{F}$) and the 3*d* states of Co (Ni), forming the band at the energy range −4–2 eV split into bonding and antibonding subbands. The minority spin 3*d* bands (down arrow) in both compounds are almost entirely occupied. Upon the Co/Ni substitution, the band only narrows slightly and shifts minutely toward the Fermi level.

Additional 3*d* electrons contributed by Ni atoms populate only the majority spin 3*d* bands. The partially populated antibonding majority spin states in the Gd_0.375_Tb_0.625_Co_2_ get fully occupied in the Gd_0.375_Tb_0.625_Ni_2_. In effect, the majority spin 3*d* band in the second compound shifts toward higher binding energies and becomes slightly narrower. It is worth noting that the bands of 3*d* states with opposite spin directions in Gd_0.375_Tb_0.625_(Co_0.5_Ni_0.5_)_2_ are almost symmetrical, which explains the vanishingly small magnitude of ${\bar{\mu }}_{Ni}$ appearing in the compound.

**Figure 17:**
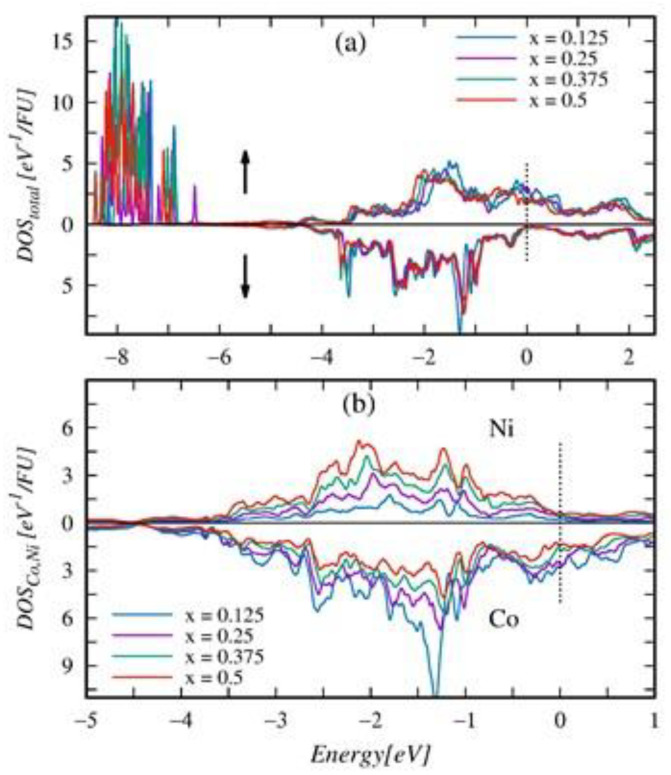
Spin resolved the total density of states *DOS_total_* (**a**), and Ni and Co contributions to the DOS (**b**) in the Gd_0.4_Tb_0.6_(Co_1−x_Ni_x_)_2_ series. For a detailed description, see the caption of Figure 16.

The variation of the calculated band structure upon Co/Ni substitution in the Gd_0.4_Tb_0.6_(Co_1−x_Ni_x_)_2_ series is displayed in Figure 17a. As concern the minority spin 3*d* band, besides a slight shift toward the Fermi level, the changes are negligible. The apparent changes occur in the majority spin bands DOS, in which the dominant peak shifts toward higher binding energy by almost 0.5 eV. The magnitude of the shift is close to that displayed by experimental spectra (Figure 13a). Figure 17b shows the variation of separated contributions of constituent Co and Ni atoms to the 3*d* band upon increasing Ni concentration. As expected, the amplitude of co-contribution decreases while that of Ni increases with the growing Ni content. However, detailed inspection indicates that upon Co/Ni substitution, the Co3*d* states shift minutely toward the Fermi level when the dominant peak of the Ni contribution shifts noticeably in the opposite direction. The last effect could explain the observed shift of maximum intensity of the XPS spectra, related to the 3*d* band, with rising concentration of Ni in Gd_0.4_Tb_0.6_(Co_1−x_Ni_x_)_2_ series.

## 3. Methods and Materials

The Gd_0.4_Tb_0.6_(Co_1−x_Ni_x_)_2_ samples x = 0.00, 0.05, 0.10, 0.15, 0.50, 0.80, 1.00 were prepared by arc melting method from high purity elements (99.99% purity) under argon atmosphere. An excess of 1% wt. of gadolinium and terbium was added to overcome weight losses during the melting. The samples have been re-melted several times to obtain the homogeneity of prepared compounds. Afterward, the as-cast samples were wrapped in tantalum foil, placed in a quartz tube, and annealed at 800 °C for two weeks. The crystal structure was determined by the X-ray diffraction technique using the XRD diffractometer Empyrean (PANalytical, Malvern, UK). The measurements were performed at room temperature with Cu K_α_ source and 2*θ* changing from 15 to 140 degrees. All magnetic measurements were carried out using the SQUID magnetometer MPMS XL–7 (Quantum Design, San Diego, CA, USA) in the temperature range from 2 K to 350 K–400 K under a magnetic field up to 7 T. The electronic structure of the investigated compounds was studied using the XPS method. The XPS spectra were obtained with monochromatized Al K_α_ radiation (*hω* = 1486.6 eV) at room temperature using PHI 5700/660 physical electronics spectrometer. All spectra were measured immediately after breaking the sample in a 10^−9^ Torr vacuum. The breaking in the high vacuum resulted in clean surfaces free of oxygen and carbon contamination. Electrical resistivity was carried out by the PPMS system. The samples were cut into rectangular shapes with dimensions of 1 × 1 × 3 mm. The measurements were performed in the temperature range of 2 K–350 K.

The ab initio electronic structure calculations for selected concentrations of Gd_0.4_Tb_0.6_(Co_1−x_Ni_x_)_2_ were performed using the FP-LAPW method [37] implemented in the WIEN2k computer programs [38]. In the present investigations, we applied the same approach and computational setup used in calculations for the reference Gd_0.4_Tb_0.6_Co_2_ compound [6].

We followed the supercell approach to simulate the fractional concentration of Co-Ni elements in Gd_0.4_Tb_0.6_(Co_1−x_Ni_x_)_2_ alloys. We adopted the superstructure Gd_3_Tb_5_Co_16_ applied in [6] in the present calculations as a base supercell. The concentrations *x*_Ni_ = 0.125, 0.25, 0.375, and 0.50 were simulated using superstructures Gd_3_Tb_5_Co_16-n_Ni_n_ where, respectively, 2, 4, 6, 8 Co atoms were replaced by Ni ones at selected sites. We are aware that for each considered concentration, there is an enormous number of Co-Ni configurations. However, we assumed that a single reasonably selected configuration (not of cluster shape) can reproduce essential features of the electronic structure of Gd_0.4_Tb_0.6_(Co_1−x_Ni_x_)_2_ alloys.

## 4. Conclusions

The new class of the Gd_0.4_Tb_0.6_(Co_1−x_Ni_x_)_2_ intermetallic compounds with the Laves phase structure of C15 type was synthesized. Structural, electronic, and magnetic properties were studied using experimental and theoretical methods.

The experimental and theoretical studies (ab initio and MFT) have shown that the magnetic moments of the R and 3*d* subnets align oppositely. With increasing nickel content, the values of 2 *μ*_3*d*_ decreased. Values of Curie temperatures determined by different methods were consistent and diminished with growing nickel content too. Arrott plots and Landau’s coefficients indicated a second-order phase transition in all studied compounds.

The values of the maximum entropy change ${\left|\Delta S_{M}\right|}^{max}$, obtained under the magnetic field change of 5 T, increased with growing Ni content from 4.13 [J/kgK] to 11.99 [J/kgK] for samples with *x* = 0.0 and 1.0, respectively. *RCP* parameters also grew significantly with Ni concentration.

Presented results revealed that the replacement of Co with Ni enhanced the magnetocaloric effect in the Gd_0.4_Tb_0.6_(Co_1−x_Ni_x_)_2_, making these materials more attractive in terms of potential applications in magnetic refrigeration. An additional advantage is that they have minimal losses observed in hysteresis loops.

Our studies have also shown that the substitution of Ni in place of Co significantly affects the resistive properties of investigated compounds. Due to a disorder of the compound’s crystal structure, an increase in the residual resistivity increases up to *x* = 0.50, which follows Nordheim’s rule. A similar variation of *H_c_*(*x*) and (*M*_FC_*-M*_ZFC_)(*x*) indicates that structural disorder may be responsible for magnetic properties.

Both XPS measurements and ab initio calculations revealed that Co3*d* and Ni3*d* states dominate the shape of the valence band near the Fermi level. With growing Ni contents, the observed intensity of the XPS spectra corresponding to 3*d* bands increases, and its maximum shifts to the higher binding energy. Ni doping, however, did not affect the positions and shapes of the Gd4*f* and Tb4*f* lines. Ab initio calculations confirmed the XPS picture.

## Figures and Tables

**Table 1:** Curie temperature (*T_C_*), saturation magnetization (*M_S_*)*,* and 3*d* sublattice contribution to the magnetic moment (2 *μ*_3*d*_) in the Gd_04_Tb_0.6_(Co_1−x_Ni_x_)_2_ system. In the table, the *exp.* and the *calc*. denote the experimental and ab initio results. The results denoted as *fit* were estimated using the formula *M_S_*(*x*) *=* 2.104 *x +*5.942 obtained by fitting the linear function to ab initio results for *M_S_* (R square = 0.998).

x		0.00	0.05	0.10	0.125	0.15	0.25	0.375	0.50	0.80	1.00
*T_C_* [K]		300.6	265.4	235.6	-	215.6	-	-	144.5	73.3	51.3
*M_S_* [μ_B_/fu]	*exp.* *calc.* *fit*	5.86 5.91 5.94	5.94 6.05	6.57 6.15	- 6.17 6.21	6.60 6.26	- 6.51 6.47	- 6.78 6.73	7.20 7.02 7.2	8.10 7.62	7.83 8.07 8.05
2 *μ*_3*d*_ [μ_B_/fu]	*exp.* *calc.*	2.34 2.57	2.26 -	1.63 -	- 2.26	1.60 -	- 1.93	- 1.64	1.00 1.39	0.10 -	0.37 0.24

**Table 2:** The exchange integrals J_RR_, J_RT_, J_TT*,*_ and magnetic moments of the 3*d* sublattice calculated in the framework of MFT theory (2 *μ*_3*d-MFT*_).

Gd_0.4_Tb_0.6_(Co_1−x_Ni_x_)_2_	J_RR_ [10^−23^J]	−J_RT_ [10^−23^J]	J_TT_ [10^−22^J]	2 *μ*_3*d-MFT*_ [μ_B_/fu]
*x* = 0.00	1.90	11.7	3.59	2.44
*x* = 0.05	1.90	9.34	2.55	2.88
*x* = 0.10	1.90	9.87	2.78	2.20
*x* = 0.15	1.90	9.35	2.65	2.09
*x* = 0.50	1.90	7.29	2.38	1.36
*x* = 0.80	1.46	2.26	1.25	1.18
*x* = 1.00	0.96	2.02	0.24	1.20

**Table 3:** The maximum of the entropy changes |*ΔS_M_*|^max^, *RC*, *RCP,* and *δT_FWHM_* parameters as a function of *x* in Gd_0.4_Tb_0.6_(Co_1−x_Ni_x_)_2_ compounds. All data are for the magnetic field of 5 T.

Gd_0.4_Tb_0.6_(Co_1−x_Ni_x_)_2_	|*ΔS_M_*|^max^ [J/kgK]	*RC* [J/kg]	*RCP* [J/kg]	*δT_FWHM_* [K]
*x* = 0.00	4.13	167.23	210.52	50.03
*x* = 0.05	4.26	190.79	230.06	54.06
*x* = 0.10	4.60	301.24	381.29	82.89
*x* = 0.15	5.00	372.01	535.62	114.03
*x* = 0.50	6.47	406.65	559.69	86.45
*x* = 0.80	10.07	467.81	623.84	61.96
*x* = 1.00	11.99	455.58	570.39	47.56

**Table 4:** The Curie temperatures *T_CR_* from resistivity measurements, residual resistivity *ρ*_0_, and fitting parameters from the Equation (7).

Gd_0.4_Tb_0.6_(Co_1−x_Ni_x_)_2_	*T*_CR_ [K]	*ρ*_0_ [μΩcm]	*α*	*β*	*ω*
x = 0.00	294.4	6.38	3.70∙10^−11^	9.38∙10^−6^	2.67∙10^−3^
x = 0.05	260.6	53.00	9.41∙10^−11^	1.84∙10^−5^	5.23∙10^−3^
x = 0.10	240.1	84.75	2.26∙10^−10^	3.12∙10^−5^	8.2∙10^−3^
x = 0.15	208.2	63.74	1.45∙10^−10^	1.72∙10^−5^	4.74∙10^−3^
x = 0.50	141.0	118.47	1.81∙10^−10^	3.07∙10^−5^	7.86∙10^−3^
x = 0.80	66.0	50.41	6.20∙10^−9^	1.24∙10^−4^	1.25∙10^−2^
x = 1.00	54.2	34.62	1.40∙10^−9^	1.09∙10^−4^	1.07∙10^−2^
